# 

**Published:** 1896-04-04

**Authors:** 


CADBURY'S COCOA

"The standard of highest purity at present attainable."—*Lancet.*

CADBURY'S COCOA

NO ALKALIES USED.

LIBRARY Index S. G. O.

### THE HOSPITAL

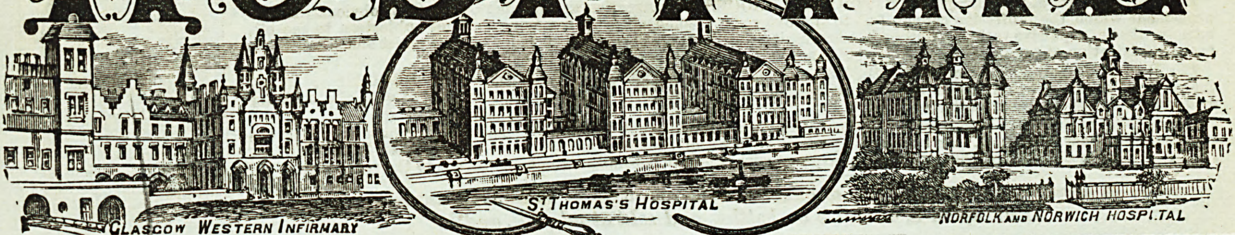

No. 497. Vol. XX.
SATURDAY, APRIL 4TH, 1896.

WITH EXTRA NURSING SUPPLEMENT.
[Registered at the General Post Office as a Newspaper for transmission in the United Kingdom and Abroad.]

PRICE TWOPENCE.
Per Annum, 10s. 6d., post free.
Monthly Parts, 6d.; post free, 8d.
Ditto per Annum, 8s. 6d., post free.

